# Efficacy of a Web-Based, Crowdsourced Peer-To-Peer Cognitive Reappraisal Platform for Depression: Randomized Controlled Trial

**DOI:** 10.2196/jmir.4167

**Published:** 2015-03-30

**Authors:** Robert R Morris, Stephen M Schueller, Rosalind W Picard

**Affiliations:** ^1^MIT Media LabMassachusetts Institute of TechnologyCambridge, MAUnited States; ^2^Center for Behavioral Intervention TechnologiesDepartment of Preventive MedicineNorthwestern UniversityChicago, ILUnited States

**Keywords:** Web-based intervention, crowdsourcing, randomized controlled trial, depression, cognitive behavioral therapy, mental health, social networks

## Abstract

**Background:**

Self-guided, Web-based interventions for depression show promising results but suffer from high attrition and low user engagement. Online peer support networks can be highly engaging, but they show mixed results and lack evidence-based content.

**Objective:**

Our aim was to introduce and evaluate a novel Web-based, peer-to-peer cognitive reappraisal platform designed to promote evidence-based techniques, with the hypotheses that (1) repeated use of the platform increases reappraisal and reduces depression and (2) that the social, crowdsourced interactions enhance engagement.

**Methods:**

Participants aged 18-35 were recruited online and were randomly assigned to the treatment group, “Panoply” (n=84), or an active control group, online expressive writing (n=82). Both are fully automated Web-based platforms. Participants were asked to use their assigned platform for a minimum of 25 minutes per week for 3 weeks. Both platforms involved posting descriptions of stressful thoughts and situations. Participants on the Panoply platform additionally received crowdsourced reappraisal support immediately after submitting a post (median response time=9 minutes). Panoply participants could also practice reappraising stressful situations submitted by other users. Online questionnaires administered at baseline and 3 weeks assessed depression symptoms, reappraisal, and perseverative thinking. Engagement was assessed through self-report measures, session data, and activity levels.

**Results:**

The Panoply platform produced significant improvements from pre to post for depression (*P*=.001), reappraisal (*P*<.001), and perseverative thinking (*P*<.001). The expressive writing platform yielded significant pre to post improvements for depression (*P*=.02) and perseverative thinking (*P*<.001), but not reappraisal (*P*=.45). The two groups did not diverge significantly at post-test on measures of depression or perseverative thinking, though Panoply users had significantly higher reappraisal scores (*P*=.02) than expressive writing. We also found significant group by treatment interactions. Individuals with elevated depression symptoms showed greater comparative benefit from Panoply for depression (*P*=.02) and perseverative thinking (*P*=.008). Individuals with baseline reappraisal deficits showed greater comparative benefit from Panoply for depression (*P*=.002) and perseverative thinking (*P*=.002). Changes in reappraisal mediated the effects of Panoply, but not the expressive writing platform, for both outcomes of depression (ab=-1.04, SE 0.58, 95% CI -2.67 to -.12) and perseverative thinking (ab=-1.02, SE 0.61, 95% CI -2.88 to -.20). Dropout rates were similar for the two platforms; however, Panoply yielded significantly more usage activity (*P*<.001) and significantly greater user experience scores (*P*<.001).

**Conclusions:**

Panoply engaged its users and was especially helpful for depressed individuals and for those who might ordinarily underutilize reappraisal techniques. Further investigation is needed to examine the long-term effects of such a platform and whether the benefits generalize to a more diverse population of users.

**Trial Registration:**

ClinicalTrials.gov NCT02302248; https://clinicaltrials.gov/ct2/show/NCT02302248 (Archived by WebCite at http://www.webcitation.org/6Wtkj6CXU).

## Introduction

Major depressive disorder is a debilitating and costly illness. In the United States alone, depression affects as many as 6.6%-10.3% of the population each year [1,2] and creates a huge economic burden, costing tens of billions of dollars [3]. To address a problem of this magnitude, innovative solutions are needed. Self-guided treatments, such as those delivered via the Web, show promise [4] and have the potential to reduce many of the practical and emotional barriers that typically prevent depressed individuals from seeking traditional psychotherapy [5]. In practice, however, many self-guided interventions suffer from high attrition rates and low levels of engagement. A recent review of self-guided, Web-based treatments found a median completion rate of 56% [6]. Open trials show even higher rates of attrition [7]. Low levels of engagement can be especially problematic and might be one of the reasons that self-guided treatments produce smaller gains than supported methods [4].

To address problems related to engagement and adherence, self-guided treatments can be augmented with external support from clinicians or coaches. Mohr et al, for example, found greater adherence to a self-guided depression intervention when participants were provided weekly 5-10 minute phone calls from an assigned coach [8]. While this approach holds promise, its ability to scale widely may be limited. Potential barriers to access include cost, availability of coaches, and scheduling logistics. Further, many individuals seek out Web-based treatments as an alternative to interacting with a clinician and may not be comfortable seeking support from other professionals [9], even trained coaches. Ideally, individuals should be intrinsically motivated to engage with intervention technologies on their own, without prompting from outside clinicians, coaches, or researchers.

Online peer support networks are extremely popular and are known to naturally engage users. Indeed, Horrigan reports that over 84% of American Internet users have visited an online community group at least once [10]. Anonymous peer-to-peer support apps also attract a wide audience. *Whisper,* the anonymous, confessional peer-to-peer app, attracted well over a billion page views a month in 2014 [11]. However, there remains a paucity of rigorous, controlled studies on the efficacy of online support groups and peer-to-peer support apps for mental health [12]. Future work is needed to determine whether these platforms are as helpful as they claim to be. For some individuals, unmoderated Internet support platforms may actually be detrimental. For example, Kaplan et al showed that individuals who participated frequently on unstructured, online mental health forums reported greater psychological distress over time [13]. Mixed findings with regard to discussion forums and peer-to-peer support apps are not surprising given the lack of oversight on the content provided in these resources.

Still, there may be a way to adapt these platforms, creating peer-to-peer interactions that are structured and moderated to reinforce evidence-based clinical techniques. It may be possible, for instance, to create an intervention that is as engaging and personalized as typical peer-to-peer platforms, while still providing the therapeutic content found in self-guided, clinical programs.

The aim of this paper is to introduce such a system, outline its design and its putative benefits, and evaluate its potential to reduce depression systems and foster engagement. In this paper, we present *Panoply—*a peer-to-peer platform that provides cognitive reappraisal and socioaffective support, anytime, anywhere. In lieu of clinician oversight, Panoply coordinates supportive reappraisals from online crowd helpers, all of whom are trained on demand, as needed. Panoply incorporates recent advances in crowdsourcing and human computation to ensure that interactions are timely [14] and vetted for quality [15].

While Panoply incorporates many novel design features, the overall user experience was built to resemble existing peer support apps: users can post content, respond to others, and get notifications when new interactions have taken place. These interactions provide natural triggers for engagement and are designed to bring users back to the platform again and again [16]. As with the most successful peer-to-peer apps, Panoply is also aligned with how individuals typically engage with technologies today. Users are increasingly likely to “snack” on apps, visiting them frequently, but in short bursts [17,18]. Therefore, instead of bundling app content into long weekly sessions that require repeated, lengthy periods of sustained attention, Panoply accommodates multiple levels of commitment. It offers tutorials and other didactic exercises, but all the content is self-contained in short, bite-sized chunks. Everything can be absorbed piece-meal, if necessary, without requiring extended time commitments on the part of the user. Taken together, these design choices highlight the importance of adapting interventions to current norms of technological interaction and consumption, rather than defaulting to holdovers from face-to-face therapy sessions or “psychological skeuomorphs” [19]. Panoply is also interactive, personal, social, and supportive and therefore includes design features that have been recommended for building engaging Web-based cognitive-behavioral therapy (CBT) interventions [20].

The primary therapeutic approach behind Panoply draws from CBT. Much of CBT’s efficacy relies on teaching people compensatory skills [21], and research supports that cognitive skills are an important mediator of symptom change [22]. A critical skill taught in CBT for depression is cognitive reappraisal—an adaptive emotion regulatory technique that involves reinterpreting the meaning of a thought or situation to change its emotional trajectory [23]. Cognitive restructuring, a form of reappraisal, is one of the most common components in Web-based treatments for depression [24-27]. On Panoply*,* users are taught reappraisal skills and are trained to think more flexibly and objectively about the stressful events and thoughts that upset them. They learn these techniques experientially, in relation to their own day-to-day problems and negative self-beliefs. They also learn by acting as respondents in the system and applying these techniques to other people. Users do not simply consume reappraisal assistance passively, they actively provide it to others as a way to rehearse and practice this technique, over and over.

While some elements of the Panoply design have been described and analyzed elsewhere [28,29], this paper is the first to introduce the complete peer-to-peer design and assess its effects within a randomized controlled trial design. In this paper, we examine the hypotheses that repeated use of this platform will reduce depression symptoms and that the social, interactive design will promote engagement. We also examine whether reappraisal mediates changes in depression symptoms and perseverative thinking for the Panoply and expressive writing platforms.

## Methods

### Study Design and Participants

We conducted a parallel-arm randomized controlled trial (RCT), assigning participants to either the Panoply intervention or an active control intervention (online expressive writing). The study was approved by the MIT Committee on the Use of Humans as Experimental Subjects (ref. no. 1311006002). Recruitment took place between April and June 2014. To be eligible for the trial, participants needed to be native English speakers between the ages of 18 and 35. This age range was selected because users in this age group are more likely to have experience with anonymous, social messaging platforms, and this study sought to find initial support for this platform rather than investigating widespread implementation.

Participants were recruited from various universities, Internet websites (craigslist, research portals), and through social media channels (Facebook, Twitter). Participants signed up on the Web, by submitting their emails on the study recruitment website. The study was advertised as an opportunity to try a new Web-based stress reduction app. It was open to the general public and depression status was not an inclusion criterion. Depression was not mentioned in any of the recruitment materials. Participants were not paid directly for participation in the study. Instead, all participants who completed the baseline and follow-up assessments were offered a chance to win an iPad Mini (valued at US $300). Use of the platform was not a factor for being eligible to win the iPad Mini.

### Procedures

Participants who submitted their emails were assigned unique, anonymous study IDs and were randomized to condition on a 1-to-1 ratio. Randomization occurred prior to any screening procedures and before participants received descriptions of their assigned intervention in the consent form. Obtaining consent after randomization can increase the probability of individuals participating in a research study [30] and has better generalizability to real-world settings in which a platform like Panoply would be used. This approach also prevented our control participants from feeling unmotivated, simply because they felt they had been assigned a less exciting, less social app. With this approach, we were less likely to encounter the “resentful moralization problem”, which is a bias that can occur when consent is provided prior to randomization [31,32]. However, this approach might result in more attrition in the stage immediately following randomization as users at this point have expressed only preliminary interest in this study. When barriers to entry are low in studies of Internet interventions, dropout, especially early on, might be very high, possibly biasing results when traditional methods of dealing with missing data are applied [33]. Typically, intent-to-treat analysis examines all those who are randomized, but that might be inappropriate when randomization is conducted prior to consent. As such, our study analyzed individuals who were consented.

Randomization and email correspondence were automatically coordinated through scripts we wrote in the Python programming language. After completing the consent form and baseline assessments, participants were asked to use their study IDs to create an anonymous account on their assigned platform. They were told to use the app for at least 25 minutes per week, for 3 weeks. To best approximate real usage with an unmoderated app, participants were not given any further instructions about how to use their assigned system. Instead, participants were told to use the app in ways that best fit their schedules and interests. Participants in both groups received four automated emails throughout the study reminding them to use their assigned app. After 3 weeks, participants were emailed a link to the follow-up assessments. The online assessments were hosted by SurveyGizmo and required a unique study ID to log in. This prevented multiple submissions. Incomplete survey data were not included in the analyses.

**Figure 1:**
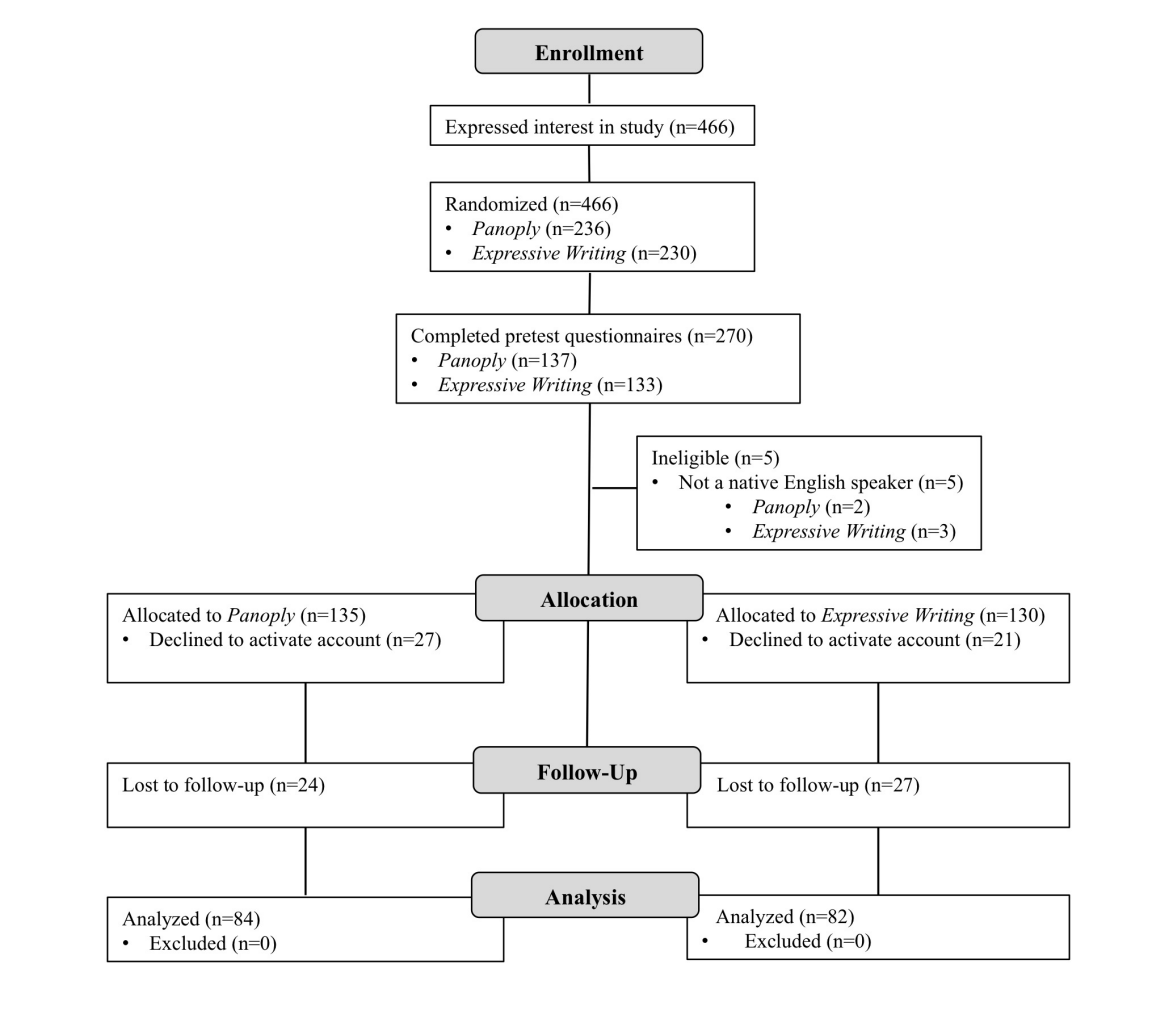
CONSORT flow diagram.

As shown in the CONSORT diagram (Figure 1), 270 individuals completed the online consent form and baseline questionnaires. Five were excluded from the study after consenting because they reported being non-native English speakers. See Multimedia Appendix 1 for the CONSORT checklist [34].

A total of 217 individuals activated an account (Panoply*=*108, expressive writing=109). Of these, 166 (76.5%) completed the follow-up questionnaires. No significant differences existed in the rates of dropout between the control or treatment interventions at any stage of the study.

Three individuals reported dropping out prematurely because they were not truly interested in a stress reduction app but wanted to explore the new technology. The social media advertisements were broadcast from MIT’s Media Lab, which has a reputation for high-tech innovation, and it is likely that this recruiting channel attracted tech-curious individuals who were not actually in need of an intervention. It is likely that others dropped out for similar reasons. Other reasons for dropout included not having enough time to adhere to the recommended 25/min per week guidelines (n=2), being out of town (n=1), or not having reliable access to a desktop computer (n=1). The remaining 41 individuals who did not activate an account could not be reached for comment and did not respond to our emails. Those who activated an account but did not complete follow-up assessments (n=51) could also not be reached for comment, despite three separate attempts to reach them by email.

Though all study procedure emails were automated, participants could email the experimenters directly during the study if they needed clarifications about the procedures or if they had technical difficulties using their assigned app. To be able to answer specific questions about either the control or treatment app, experimenters were not blind to the random assignment of participants. However, during the course of the study, only four participants emailed the experimenters to request technical support or procedure clarification.

### Interventions

#### Treatment Group (Panoply)

##### Overview

**Figure 2:**
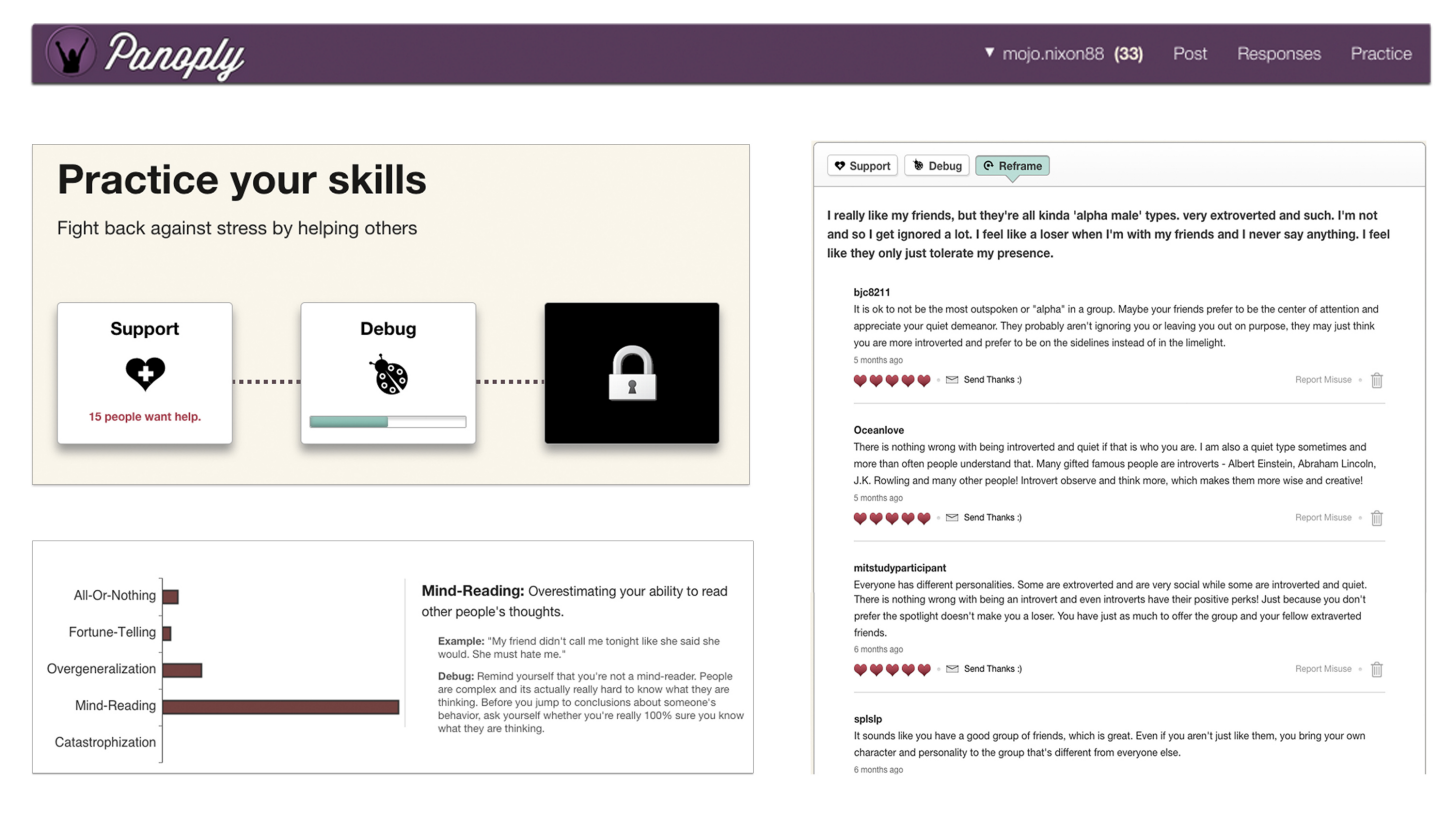
Screenshots from the Panoply platform, illustrating the tutorial panel, the debug dashboard, and the reframe responses.

Panoply is a peer-to-peer, Web-based cognitive reappraisal program developed at the MIT Media Lab. Unlike other Web-based psychoeducation platforms (eg, [35,36]), Panoply does not rely on static, didactic content to teach therapeutic techniques. Rather, the Panoply platform is a dynamic, social, and interactive platform. Panoply users can post content, respond to others, receive responses, and get feedback on their performance (Figure 2). Panoply also offers additional structure, training, and moderation to ensure that all interactions on the site are aligned with evidence-based therapeutic techniques. In the sections that follow, we examine the three core behaviors that occur on Panoply: posting content, responding to others, and receiving responses.

##### Posting Content

The initial activity on Panoply involves posting short descriptions of negative thoughts and situations (500 characters maximum). When posting, users are asked to first describe a stressful situation, using one to two sentences. Next, they are asked to record any automatic negative thoughts they might have about the situation. A short tutorial helps first-time users understand the difference between negative situations and the automatic negative thoughts associated with them.

**Figure 3:**
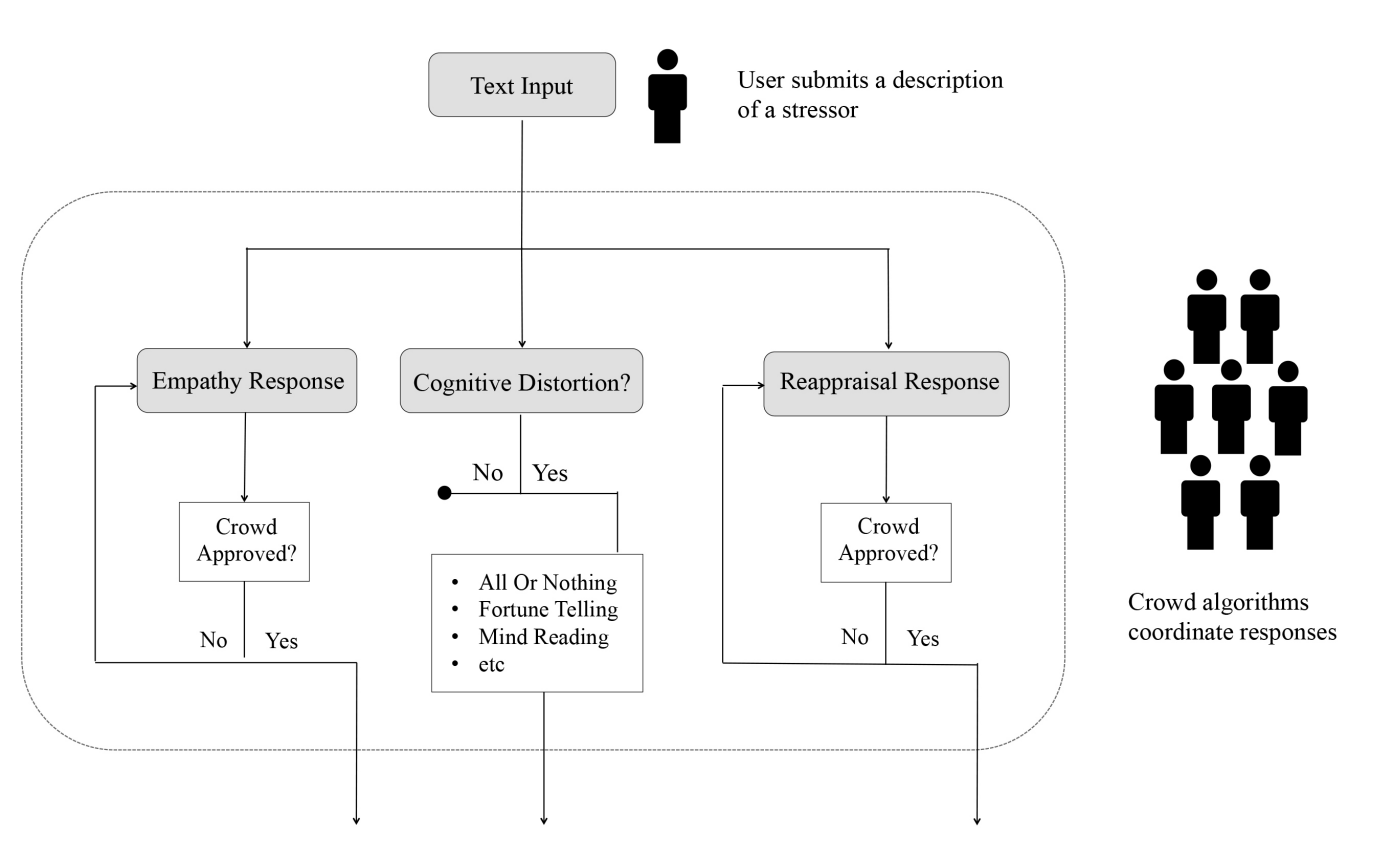
Multiple sets of crowd workers are coordinated to compose and curate responses on the system. The cognitive distortions are identified and labeled, but not subject to crowd review.

Once a user posts on Panoply, a sequence of crowdwork is automatically set into motion (Figure 3). First, crowd helpers from Amazon’s Mechanical Turk service (MTurk) are hired to review each post. Any post that contains offensive material, off-topic content, or language related to self-harm is excluded from the system. In the case of language related to self-harm, an automated email is immediately sent to the author of the post. The email includes links to mental health resources and reminds the poster that the system is a self-help tool, not to be used for crisis-related situations. Once a post is approved, it is automatically delivered to several sets of trained respondents.

##### Responding to Others

Before being given a chance to respond to the post of a peer, each respondent is trained to use a specific therapeutic technique. Respondents are taught to (1) offer empathy, (2) identify cognitive distortions, or (3) help users reframe negative situations in ways that are more positive and adaptive. Also, responses are vetted by other crowd helpers before being returned to the person who made the original post. If a response is deemed inappropriate or abusive, it is immediately discarded. All of the aforementioned interactions are coordinated entirely through Panoply’s automation. The user needs to only submit their post to start this sequence of crowd work.

Respondents in our study were a mixture of other Panoply users and paid workers from MTurk. Workers from MTurk are used for several reasons. First, because Panoply is not yet a large, peer-to-peer system, MTurk provides a temporary stand-in for a large user base, helping ensure that users receive lots of responses extremely quickly (the median response time on Panoply was 9 minutes). Second, MTurk workers can be hired at marginal cost to efficiently moderate content ($0.01) and composes responses ($0.10-0.14) on the system. In our study, the average weekly cost per Panoply user was US $1.04. This could eventually drop to zero. Indeed, if Panoply were to attract a large and active user base, MTurk workers would probably not be needed.

Training for both MTurk workers and Panoply site users occurs on demand, as needed, and involves short, 3-5 minute training modules. Users are introduced to a specific therapeutic technique, are shown positive and negative exemplars of responses, and complete an interactive quiz to assess comprehension. After successfully completing the training, users are given the opportunity to practice the technique by responding to a post from a real Panoply user.

MTurk workers receive a small amount of payment for each response they compose. Panoply users, by contrast, contribute for free. They are told that each time they respond to others they get to practice techniques that are important for managing stress and negative emotions. They are reminded that teaching others can be an exceptionally great way to learn. This concept, a form of peer-based learning, has been studied at length in pedagogical research [37]. To our knowledge, this peer-based learning approach has rarely, if ever, been used in the context of Web-based depression interventions.

Responses on Panoply fall into three categories: support, debug, and reframe. These categories were drawn from evidence-based practices for depression and have been examined on earlier versions of the Panoply system [28]. Support responses offer emotional support and active listening. Debug responses help users identify and dispute cognitive distortions (“bugs”). Reframe responses offer alternative, more positive ways of thinking about the stressful situation. Respondents are not asked to use any one particular reappraisal strategy but instead are given a bulleted list of tactics to consider in case they need inspiration. These prompts were culled from reappraisal taxonomies and strategies cited in the emotion regulation research literature [38,39].

##### Receiving Responses

The response panel on Panoply features a button-based navigation bar that lets users switch between the three types of responses generated by the crowd. When a user visits the response panel, support messages are displayed first by default and are listed in a newsfeed format (the most recent appearing at the top). Users can rate these responses as they appear in the interface. If particularly moved, users can compose a short note, thanking the respondent for their contribution.

The debug section features a graphical dashboard of cognitive distortions (“bugs”) identified by the crowd. The dashboard includes personalized suggestions that describe how to restructure the specific distortions that were observed by the crowd.

The reframe section features short messages, displayed in the same newsfeed format as the support responses. Unlike the other response categories, however, the reframes are not revealed outright. When users get notified of new responses in this category, they are not able to view them initially. Instead, users must compose a reappraisal for themselves before they can access responses from the crowd. The hope is that users will be naturally moved to complete reappraisals for themselves because they know that doing so unlocks interesting new social content.

#### Control Group (Online Expressive Writing)

**Figure 4:**
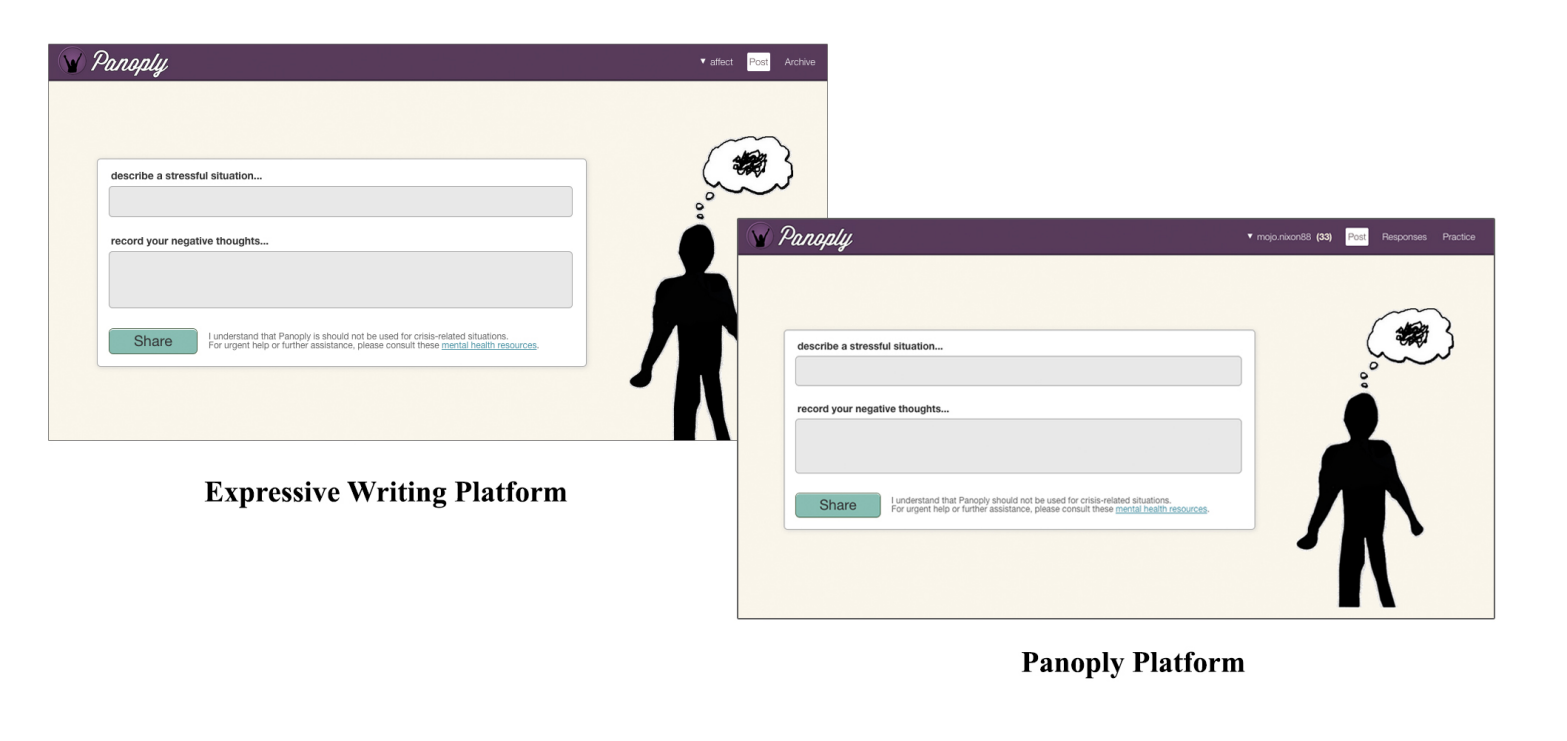
The control and treatment platforms were matched on non-specific factors, including visual design, user interface design, and logotype.

The visual and interface design for the control condition was built to mirror the Panoply intervention. The instructions for describing stressful situations and negative thoughts were exactly the same (Figure 4). However, users in this condition did not receive feedback from the crowd and were not given the opportunity to provide feedback for others. We did not expect this condition to be inert because writing expressively about negative experiences is a well-studied and efficacious intervention it its own right and can help reduce depression symptoms [40]. A meta-analysis has found that expressive writing in various formats can improve physical and psychological health outcomes [41]. Our writing condition was a useful control because although it matched Panoply on nonspecific factors (eg, Web design, user registration, composing negative thoughts), it did not contain reappraisal training or crowdsourced interactions. However, it did allow users to engage in a similar process of entering content, thus allowing for comparisons that control for the effects of being online and putting feelings into words.

### Usability Testing

Both platforms underwent several usability studies, both in the lab and over the Web. For lab-based studies, an experimenter was present at all times and the sessions were moderated using techniques such as “concurrent thinking aloud”, “retrospective thinking aloud”, and “retrospective probing” [42]. MTurk workers were also recruited online to help identify potential design flaws. These studies helped identify user experience issues and points of confusion around site navigation and other user interface components (eg, buttons, links), enabling us to refine the usability before the RCT was conducted.

### Outcome Measures

Participants completed assessments online, both at baseline and at 3-weeks’ follow-up. The primary outcome measure was the Center for Epidemiologic Studies Depression Scale (CES-D) [43], a 20-item self-report scale that assesses symptoms of depression. Secondary outcome measures included reappraisal frequency, as assessed by the Emotion Regulation Questionnaire - Reappraisal (ERQ-R) [23], and maladaptive rumination, as assessed by the Perseverative Thinking Questionnaire (PTQ) [44].

#### Center for Epidemiologic Studies Depression Scale

The CES-D [43] is a 20-item self-report scale that assesses symptoms of depression. Respondents are asked to indicate the extent to which they have felt various depression symptoms over the past week. The questions address symptoms such as loss of appetite, depressed mood, and feelings of loneliness. A score of 16 or higher suggests a high level of depression and is often used as a cut-off to determine clinically relevant symptoms.

#### Emotion Regulation Questionnaire – Reappraisal

The ERQ is a 10-item questionnaire that assesses individual differences in the habitual use of two emotion regulation strategies: cognitive reappraisal and expressive suppression. It produces scores for both reappraisal and suppression. For the purposes of this study, we analyzed only the reappraisal scores. Reappraisal is considered an adaptive regulatory strategy and is associated with positive psychological functioning, including increased positive affect, well-being, and interpersonal functioning [23].

#### Perseverative Thinking Questionnaire

Depressive rumination is a cognitive style that involves repetitive elaboration of the symptoms and causes of distress. In essence, it is an unproductive form of reappraisal. Instead of recasting a situation in ways that lead to positive recontextualizations and problem solving insights, depressive rumination mires individuals in circular reinterpretations that serve only to magnify distress. Rumination is considered a risk factor for depression and suicide and is thought to play a causal role in the development and maintenance of depressive illness. The PTQ [44] is a 15-item scale that assesses three components of rumination: its repetitiveness, its unproductiveness, and its tendency to capture mental capacity.

#### User Experience Questionnaire

To assess engagement, we administered an online version of the User Experience Questionnaire (UEQ) [45]. This self-report measure examines a product’s ability to promote an engaging user experience. The UEQ includes 26 pairs of contrasting attributes (eg, “pleasant vs unpleasant”; “motivating vs demotivating”) that are ordered along a 7-point bipolar Likert scale. For each test item, the Likert points represent gradations between the two labels. Selections indicate which of the two labels applies best to the technology being assessed.

#### Behavioral Activity Levels

We also examined activity levels on both the treatment and control apps. These data were logged automatically by our server and by Google Analytics.

Adherence rate for module completion, while a common metric for many online mental health interventions, does not apply to Panoply or the expressive writing intervention. Neither platform utilized the kind of psychoeducation modules that are typically found in Web-based CBT interventions. Further, Donkin et al recently examined the relationship between various engagement metrics and outcome in an online intervention for depression and found that the total number of modules completed was less important than the level of activity observed per login [6].

Therefore, for behavioral measures of engagement, we examined usage level and assessed the amount of activity observed per login. Specifically, we compared the average number of words written by individuals in the treatment versus control group. This is a useful metric because both the Panoply and the expressive writing interventions involve a considerable amount of writing. Writing is the only task activity one can perform on the expressive writing task. Similarly, with the exception of the “debug” exercise and the training modules, all activities on Panoply require writing. A Python script was used to compute the number of words submitted by individuals in the treatment and control conditions.

To assess the frequency and duration of logins, “sessions” data from Google Analytics were used. A “session” is defined as the period of time a user interacts with a site. Google sessions expire as soon as a user is inactive for 30 minutes.

### Analytic Plan

We used chi-square and *t* tests to evaluate whether randomization yielded equivalent demographic and symptom characteristics at baseline for the treatment and control groups. Chi-square tests were also used to compare rates of dropout between the two interventions. For engagement analyses, *t* tests were used to compare UEQ scores and word counts across the treatment and control interventions.

For psychological outcomes, we conducted a set of planned analyses to explore the overall effects of the platforms, as well as specific moderators and mediators. The stages of these analyses follow from our primary hypotheses. To reduce unnecessary multiple tests, we progressed to the next stage of analysis given significant findings at each stage. First, we explored changes within and between each condition. Our primary analyses compared the difference between the groups at post-test using linear regression models controlling for baseline levels of the dependent measure. Because Panoply was designed to teach reappraisal skills, we hypothesized that Panoply would result in greater improvements for those with deficits in this skill (as measured by reappraisal on the ERQ at baseline), and we posited that reappraisal might be a useful mechanism of action within the Panoply condition. Secondary analyses then added moderator variables as interactions within these models to explore if people with different characteristics at baseline benefited more or less from the intervention. For depression status, participants were separated into two groups based on normative values on the CES-D. Following the standard cutoff for clinically meaningful symptoms, we classified individuals scoring 16 or higher as depressed. Based on this categorization, 47 Panoply participants and 44 expressive writing participants were classified as depressed. For reappraisal, participants were dichotomized into two groups (high and low reappraisers) based on a median split. This resulted in 41 low reappraisers for Panoply and 28 low reappraisers for the writing platform. Lastly, because we believed that change in reappraisal is the key skill taught through Panoply, we explored whether reappraisal was a mediator of changes in depressive symptoms and perseverative thinking. We examined mediation using Preacher and Hayes (2008) bootstrapping procedure and SPSS macro. This procedure produces the bias-corrected and accelerated bootstrapped confidence intervals of the product of the direct pathways between condition and the mediator (a) and the mediator and the outcome (b) to estimate the indirect effect (ab).

All participants who activated an account and completed follow-up assessments were included in the analyses. Some participants, however, were lost to follow-up and were not included in the analysis of outcomes. As only two assessment time points were obtained, methods of data imputation were not used. 

## Results

### Participant Characteristics

No significant differences in baseline characteristics or dropout rates were observed between the control and treatment interventions (Table 1). The sample was 71.7% female, with a mean age of 23.7. Participants were well educated: 88.6% reported having had at least some college education and 45.8% reported having a 4-year college degree or higher.

**Table 1:** Baseline characteristics of the participants.

Characteristics		Total sample (n=166)	Treatment (n=84)	Control (n=82)	*t* or χ^2^	*P*
**Demographics**						
	Age, mean (SD)	23.7 (5.3)	23.5 (5.2)	23.9 (5.5)	-0.4^b^	.70
	Female, n (%)	119 (71.7)	62 (73.8)	57 (69.5)	0.20^c^	.66
	Higher education,^a^ n (%)	76 (45.8)	37 (44.1)	39 (47.6)	0.09^c^	.77
**Baseline scores, mean (SD)**						
	CES-D	19.0 (10.4)	19.4 (10.2)	18.6 (10.6)	0.52^b^	.61
	ERQ-R	26.4 (6.9)	26.0 (6.9)	26.7 (6.7)	0.72^b^	.47
	PTQ	47.5 (10.9)	46.8 (10.7)	48.2 (11.1)	0.87^b^	.39

^a^Equivalent to a 4-year Bachelor’s degree or higher.

^b^ *t*_164._

^c^χ^2^_1._

### Psychological Outcomes

We first examined changes in primary (depression symptoms) and secondary (reappraisal, perseverative thinking) outcomes within each condition. Table 2 displays baseline and post-intervention scores on all outcomes for each condition. Participants in the Panoply condition reported significant changes on all dependent measures, whereas those in the expressive writing condition reported significant changes in depression and perseverative thinking.

We then tested whether differences existed between the conditions at post-intervention controlling for baseline levels. No significant differences existed between the control and treatment conditions at post-test on depressive symptoms, controlling for baseline depression: *t*_164_=-0.82, *P*=.41, beta=-1.02, 95% CI -3.47 to 1.43. Similarly, no significant differences between conditions were found for perseverative thinking: *t*_164_=-0.56, *P*=.57, beta=-.82, 95% CI -3.69 to 2.05. Panoply users did, however, report significantly greater levels of reappraisal compared to users of the expressive writing condition controlling for baseline levels: *t*_164_=2.29, *P*=.02, beta=1.98, 95% CI 0.27-3.68.

Second, we wanted to assess whether Panoply was more useful for users with certain characteristics. Indeed, we observed that depression status was a significant moderator of both post-test depressive symptoms, *t*_164_=-2.28, *P*=.02, beta=-5.53, 95% CI -10.3 to -0.76, and perseverative thinking, *t*_164_=-2.70, *P*=.008, beta=-7.79, 95% CI -13.47 to -2.10. The same was true for high versus low reappraisers. Reappraisal scores at baseline were a significant moderator of depressive symptoms, *t*_164_=3.12, *P*=.002, beta=7.64, 95% CI 2.80-12.47, and perseverative thinking, *t*_164_=3.19, *P*=.002, beta=9.14, 95% CI 3.49-14.80. Participants who were depressed or low reappraisers at baseline benefited more from Panoply compared to expressive writing. The importance of depression status and reappraisal in terms of predicting who benefits, in addition to the fact that Panoply had a stronger effect on change in reappraisal than expressive writing, suggests that change in reappraisal might be an important mediator of the benefits of Panoply, so we investigated that further.

**Figure 5:**
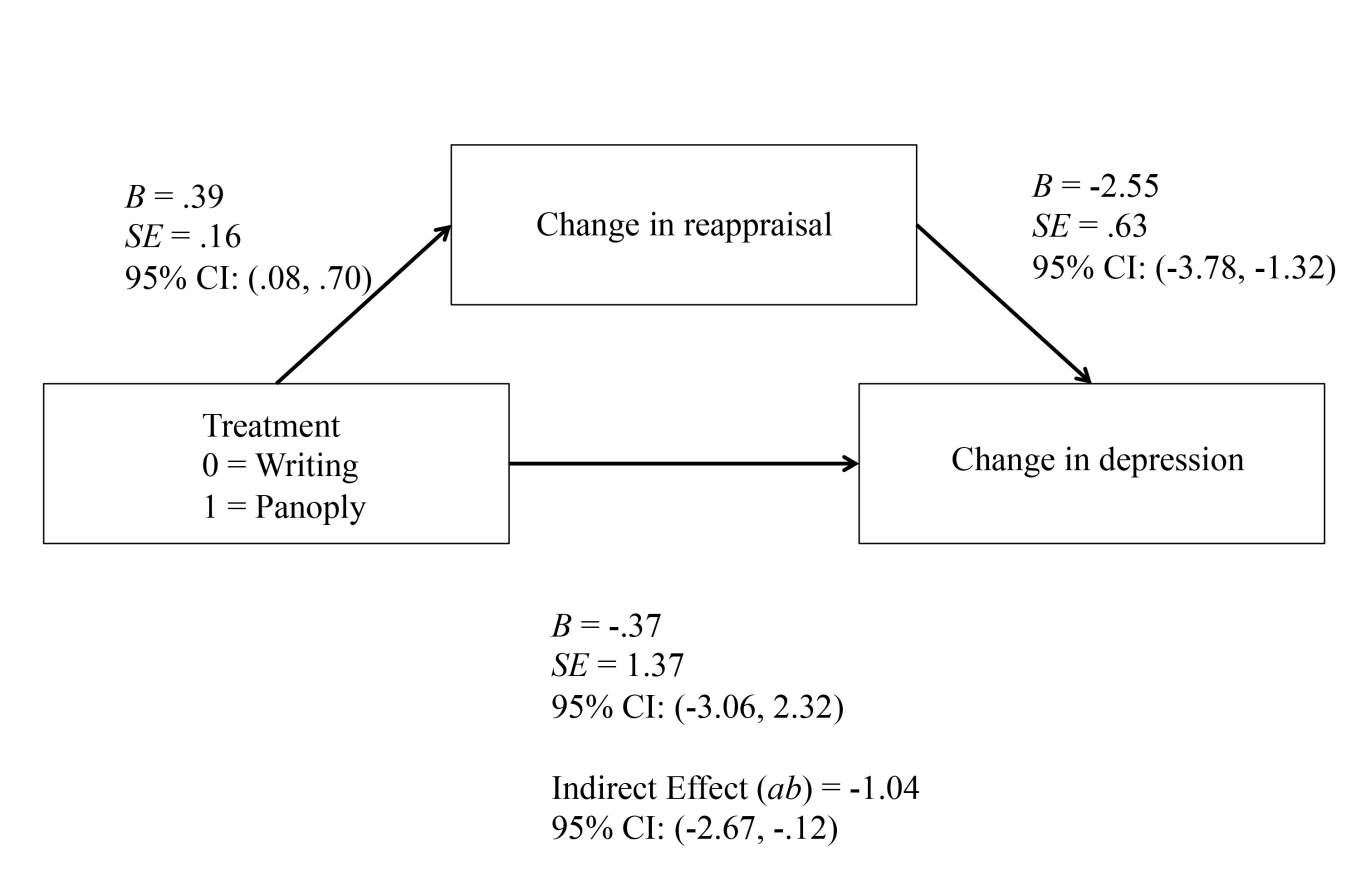
Mediation of change in reappraisal on change in depression.

Using Preacher and Hayes’ (2008) bootstrapping method, we examined whether improvement in reappraisal was a mediator of the effect of Panoply. Figure 5 displays the results of this analysis for depressive symptoms. The effect of treatment on change in reappraisal (a) was statistically significant (*B=*.39, SE 0.16, *P*=.02, 95% CI 0.08-0.70). The effect of change in reappraisal on change in depression (b) was also statistically significant (*B*=-2.55, SE 0.63, *P*<.001, 95% CI -3.78 to -1.32). The indirect effect of Panoply on changes in depression via changes in reappraisal was statistically significant (ab=-1.04, SE 0.58, 95% CI -2.67 to -0.12). These results suggest that change in reappraisal may be a specific mechanism of Panoply compared to the writing condition in reducing depressive symptoms.

**Figure 6:**
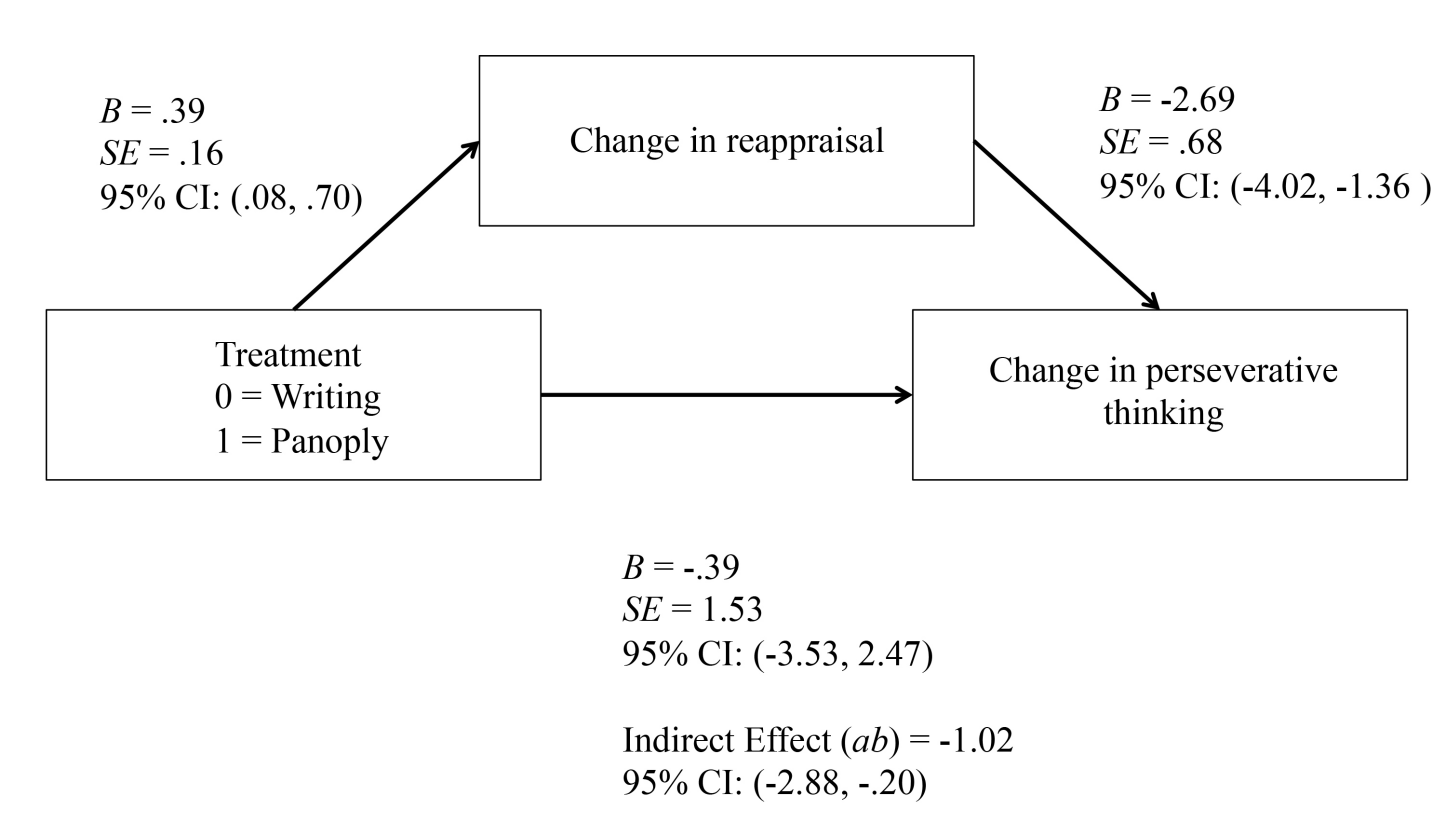
Mediation of change in reappraisal on change in perseverative thinking.

We also assessed whether change in reappraisal was a mediator of changes in perseverative thinking. Figure 6 displays the results of this analysis. The (a) pathway is the same as the previous analysis. The effect of change in reappraisal on change in perseverative thinking (b) was also statistically significant (*B*=-2.69, SE 0.68, *P*<.001, 95% CI -4.02 to -1.36). The indirect effect of Panoply on changes in depression via changes in reappraisal was statistically significant (ab=-1.02, SE 0.61, 95% CI -2.88 to -0.20). These results suggest that change in reappraisal may be a specific mechanism of Panoply compared to the writing condition in reducing perseverative thinking.

**Table 2:** Changes from baseline to post-intervention by condition.

		Pre, mean (SD)	Post, mean (SD)	Change, mean (SD)	95% CI	*T* ^a^	*P*	*d*
**Panoply**								
	CES-D	19.38 (10.16)	15.79 (9.53)	-3.60 (9.86)	-5.74 to -1.45	3.34	.001	-0.36
	ERQ-R	25.99 (6.91)	28.92 (6.22)	2.93 (6.11)	1.60 to 4.25	4.39	<.001	0.48
	PTQ	46.76 (10.70)	42.35 (11.04)	-4.42 (10.13)	-6.62 to -2.22	4.00	<.001	-0.44
**Expressive Writing**								
	CES-D	18.55 (10.60)	16.33 (10.38)	-2.20 (8.24)	-6.03 to -0.41	2.44	.02	-0.24
	ERQ-R	26.74 (6.65)	27.32 (6.78)	0.57 (6.85)	-0.93 to 2.08	0.76	.45	0.06
	PTQ	48.23 (11.11)	44.21 (13.12)	-4.02 (9.54)	-6.12 to -1.93	3.82	<.001	-0.44

^a^ *df*=83 for *Panoply* and 81 for Expressive Writing.

### Engagement Outcomes

Engagement measured by the UEQ was significantly higher for the Panoply platform (mean 137.10, SD 20.93) than the expressive writing platform (mean 122.29, SD 20.81), (*t*_164_=4.57, *D=*.71, 95% CI 1.02-0.39, *P*<.001).

There was a significant difference in activity level between conditions (*t*_164=_-4.1, *D=*.62, 95% CI 0.31-0.94, *P*<.001), with individuals in the Panoply condition writing significantly more words (mean 1013.28, SD 1145.14) than those in the expressive writing condition (mean 433.85, SD 609.59). This is striking, given that writing was not the only activity available to Panoply users. They could also identify distortions and review crowd-generated feedback—two additional activities that were used frequently but were not included in the word count metric.

Descriptive statistics from the Google session data revealed that users in the Panoply condition logged 21 sessions over the 3-week deployment on average. Their average time per session was 9 minutes and 18 seconds per session. By comparison, users in the expressive writing condition logged an average of 10 sessions, spending an average of 3 minutes and 10 seconds per session. Thus, the Panoply group averaged a total of over 195 minutes over the course of the study, a considerably longer amount than was suggested (75 minutes). Inferential statistics could not be computed, because the Google session data were not revealed at the level of the user.

### Usage Patterns

In addition to directly comparing measures of engagement between the treatment and control groups, we also captured general usage patterns for each platform (see Table 3). For the purposes of this analysis, we included everyone who activated an account and accessed the site (N=214), even if they did not return to complete follow-up assessments.

Of note is the fact that individuals assigned to the writing condition submitted considerably more posts than those in the Panoply condition. There are several possible explanations for this. First, those in the writing condition had only one task to do. Their attentions were never diverted elsewhere; the number of posts they wrote reflects their entire contribution to the site. By comparison, those in the Panoply condition could divide their time between submitting posts, responding to others, and reviewing responses from the crowd. Second, those in the Panoply condition may have been more tentative about submitting posts, simply because they had an audience. To the extent that submitting posts was therapeutic, participants in the Panoply condition, on average, received less than half the dose of those in the writing condition. Future designs of Panoply should offer additional incentives for users to post more frequently if this activity is determined to be helpful. For example, users might be given the option to record negative thoughts privately, should they want to reframe their thoughts on their own, without any input from the crowd.

**Table 3:** Average usage patterns for the Panoply and expressive writing apps for 3 weeks.

Activity		Group	Mean frequency (SD)
**Posts**			
		Panoply	2.72 (2.76)
		Writing	6.62 (8.76)
**Responses**			
	Support	Panoply	8.94 (11.95)
	Debug	Panoply	10.82 (13.72)
	Reframe	Panoply	5.99 (8.71)

### Adverse Events

One study participant composed several troubling and off-topic posts on the Panoply platform. MTurk workers detected this behavior and an automated email was sent, reminding the participant that Panoply is a self-help tool, not a formal mental health resource. Links to mental health resources were also emailed automatically to this participant. After consulting with the MIT IRB, we decided to prevent this participant from posting any further content. This individual was not withdrawn from the study, however, and was still allowed to compose responses to other Panoply users. None of the responses this individual made to others were flagged as off-topic, malicious, or otherwise inappropriate.

## Discussion

### Psychological Outcomes

Overall, participants allocated to the Panoply platform received greater clinical benefits than those assigned to the writing task. While the two platforms did not diverge significantly at post-test with respect to depression or perseverative thinking, Panoply users reported significantly high levels of reappraisal. Further, follow-up analyses suggest that individuals with elevated depression symptoms stand to benefit more from a platform like Panoply than from expressive writing. Panoply produced significantly less depression and perseverative thinking for individuals with high depression scores at baseline. A similar pattern was found for individuals who scored low on reappraisal at baseline.

As opposed to many Web-based interventions for depression that attempt to teach a variety of strategies drawn from CBT [8,24-27], Panoply specifically targets cognitive reappraisal. A benefit of this approach is that it allows testing the specific mechanism of change corresponding to that behavior change principle. Follow-up analyses suggest that the benefits accrued from Panoply appeared to be mediated by changes in reappraisal skills. Specific, targeted interventions such as Panoply*,* with a well-understood mechanism of action, can offer personalized treatment approaches, providing a powerful resource for those who do not typically use reappraisal skills to regulate emotions.

### Engagement Outcomes

Panoply was engineered to be an engaging mental health intervention. The final system incorporated many features that were specifically designed to enhance user experience. Indeed, it was hoped that many users would find the crowdsourced interactions particularly novel, motivating, and exciting. Therefore, it was hypothesized that Panoply would score higher on both self-reported user experience and behavioral measures of activity, relative to the expressive writing condition. These hypotheses were confirmed.

### Limitations

There are several methodological shortcomings that limit the generalizability of this study. First, the duration of the study was extremely short (3 weeks). While it is encouraging that Panoply managed to confer benefits in this short time, it is unclear how enduring these improvements might be. Additional long-term follow-ups are needed. Also, the study was limited to individuals aged 18-35. Additional research is needed to examine whether similar effects might be observed for other populations of users. Our sample was also largely female, and future studies will need to seek a more balanced gender distribution. Moreover, while expressive writing was a useful control comparison for many reasons, future studies should compare Panoply to more traditional, Web-based CBT programs.

In addition to these methodological limitations, there were some design shortcomings. For the purposes of this study, Panoply was built to target reappraisal skills first and foremost. While this design enabled us to test specific hypotheses about how reappraisal might mediate therapeutic outcomes, it may have limited the therapeutic potential of the platform. Future versions of this type of platform should address other techniques besides just cognitive reappraisal. For instance, Panoply could be extended to address some of the behavioral components of CBT. Behavioral interventions from positive psychology could also be incorporated in future versions, as described by Morris & Picard [29]. Finally, all interactions with Panoply were made through a Web browser, optimized for use on a laptop or desktop. To increase engagement, the platform could be redesigned for mobile use, to better align with contemporary technology usage patterns.

### Conclusions

In this paper, we introduced and evaluated a Web-based, peer-to-peer cognitive reappraisal platform designed to promote reappraisal and thus reduce depression symptoms. We found that repeated use of our system produced significant benefits, particularly for depressed individuals and for those who typically underutilize reappraisal strategies. Furthermore, we believe Panoply conferred benefits because it taught reappraisal skills. On the platform, users gained exposure to reappraisal by (1) receiving reappraisal assistance from the crowd and (2) by repeatedly reframing the thoughts and situations of others on the network. Our mediation analyses suggest that reappraisal helped reduce depression and perseverative thinking for the Panoply platform, but not the expressive writing platform. This supports our hypothesis that Panoply’s unique features are especially helpful for building reappraisal skills.

We also found that the platform engaged its users. Indeed, the Panoply platform inspired well over twice as much activity as the control platform. Further work is needed to assess whether these findings might extend to a wider, more diverse set of individuals. The longevity of these effects should also be examined. Measuring engagement over time is an important area for future research. For example, future studies should examine the rates at which individuals revisit intervention platforms on their own, as needed. Unlike other interventions that offer a limited amount of psychoeducation modules, our platform offers a potentially inexhaustible source of varied social content. As long as individuals continue to submit posts on the platform, there remain interesting new opportunities to practice therapeutic techniques. Interventions like ours, that can theoretically be revisited again and again, without appearing stale, could have unique benefits. Further research is needed to address these possibilities.

## Multimedia Appendix 1

CONSORT-EHEALTH checklist V1.6.1 [46].

## Abbreviations

CBT: cognitive-behavioral therapy
CES-D: Center for Epidemiologic Studies Depression Scale
ERQ-R: Emotion Regulation Questionnaire – Reappraisal
MTurk: Amazon’s Mechanical Turk Service
PTQ: Perseverative Thinking Questionnaire
RCT: randomized controlled trial
UEQ: User Experience Questionnaire
